# Associations among Maternal Trauma History, Postnatal Maternal Sensitivity, and Infant Temperament

**DOI:** 10.3390/children11030301

**Published:** 2024-03-02

**Authors:** Jennifer Lynn Hambleton, Nicki Lynn Aubuchon-Endsley, Jasmin Kurien

**Affiliations:** 1 Survey Healthcare Global, Nashville, TN 37214, USA; hambjenn@isu.edu; 2 Department of Psychology, University of Tulsa, Tulsa, OK 74104, USA; jak2034@utulsa.edu

**Keywords:** maternal, prenatal, infant, trauma, temperament, development, sensitivity

## Abstract

Women are at increased risk of trauma exposure and of experiencing prolonged posttraumatic stress. Maternal trauma exposure and associated impairment may adversely impact mother–infant interaction quality, which may in turn be associated with infant temperament difficulties. More research is needed to identify which maternal trauma predictors are most robustly related to infant temperament outcomes. The present study aimed to address this gap by examining maternal sensitivity as a mediator of relations between maternal trauma and infant temperament in a longitudinal study of a rural cohort of mother–infant dyads. Mediation via maternal sensitivity was not supported in any of the primary analyses. Greater maternal trauma exposure was found to predict greater infant regulation behavior, a finding that was in opposition to expected results and which may be explained by protective factors found within the sample. The present sample was skewed toward married, educated women who reported high social support satisfaction and low trauma-associated impairment. The findings elucidate protective factors that may mitigate adverse outcomes for both mothers and infants. Future research models should include additional maternal trauma variables (e.g., recency, type, revictimization/polyvictimization), in utero maternal cortisol exposure, maternal insensitivity/ambiguous response patterns during caregiver tasks, and analysis of the IBQ-R subscales.

## 1. Introduction

Women are twice as likely as men to meet the diagnostic criteria for posttraumatic stress disorder (PTSD) following trauma exposure, and on average, they experience symptoms longer [1]. Research suggests that socially gendered roles, such as caregiving, are positively associated with the observed elevation of trauma symptoms in women who identify as caregivers [2]. Moreover, maternal caregiving behaviors have been shown to influence early infant emotional development following birth [3,4,5]. Given women’s increased risk of trauma affecting on daily functioning, along with the psychosocially influential role that women fill to support their infants’ early development, it is crucial for researchers to examine early risk factors that may increase mother and infant vulnerability to adverse outcomes associated with maternal traumatic experiences. The perinatal period represents an ideal time point to study early outcomes in relation to maternal trauma because maternal trauma symptoms are often unresolved prior to motherhood [6]. Moreover, prenatal psychophysiological stress may affect the nature and quality of maternal–infant interactions in the postnatal period, which are also influenced by maternal biopsychosocial stress and mental health [7,8]. Therefore, the present study aims to examine relations between maternal trauma history and infant temperament and how these relations may be mediated by postnatal maternal sensitivity toward infants.

### 1.1. Trauma

According to the Diagnostic and Statistical Manual of Mental Disorders (5th Ed.) [9], traumatic events are defined as “exposure to actual or threatened death, serious injury, or sexual violence”, which may be directly experienced, witnessed in person, learned of about a loved one, or comprise repeated or extreme exposure to similar events [9] (p. 271). Additionally, the DSM-5 states that “the greater the magnitude of trauma, the greater the likelihood of posttraumatic stress disorder” [9] (p. 278). Given the notable variability in different types of traumatic events and posttraumatic stress (PTS) symptom experiences (e.g., experiences may range from no symptoms, to symptoms below a diagnostic threshold, to a PTSD diagnosis), it is important to define severity in terms of both trauma exposure and PTS (to capture a broad range of experiences outside the scope of PTSD diagnosis). Regarding trauma exposure severity, there are several contributing risk factors, which include repeated trauma exposure, event proximity, indirect versus direct exposure, and degree of harm [9]. Therefore, women who experience complex trauma (e.g., repeated exposure), close proximity, and a greater degree of harm from trauma exposure are more likely to experience more clinically elevated posttraumatic stress (PTS) symptoms. PTS symptom severity may be defined via continuums of frequency, intensity, and duration and the associated impaired functioning [1]. For example, women who were exposed to childhood trauma (CT) may report different frequency, intensity, and duration of experiences that would differentially impact functioning over time. One mother may report no longer being affected by her past CT, whereas another may experience PTS symptoms throughout her life as a result of CT (even without a PTSD diagnosis). Therefore, severity of both trauma exposure and PTS in both general and clinical populations stand to elucidate the nature of relations between maternal behaviors and potential infant outcomes.

#### 1.1.1. Childhood Trauma

Maternal CT has been shown to predict more neutral maternal affect during mother–infant interactions [8]. Recent research on maternal childhood emotional abuse and neglect revealed that maternal childhood emotional abuse predicted lower maternal sensitivity toward infants and greater dysfunction in mother–child interactions [10,11]. Additionally, maternal childhood physical abuse has been positively associated with emotionally withdrawn caregiving behavior, hostile maternal behaviors (e.g., behaviors that communicate irritation or disgust), mismatched maternal behaviors (e.g., speaking pleasantly about negative content), and negative infant affect [12]. Similarly, mothers who score lower on positive parenting demonstrated by behavioral observations of maternal behavioral sensitivity, engagement, warmth, affective sensitivity, and positive affect have infants who score lower in behavioral observations of emotion regulation [13].

#### 1.1.2. Disasters

An empirical review of perinatal health and disaster exposure (e.g., terrorist attacks, environmental and chemical disasters, and natural disasters) indicated that across studies, the severity of disaster exposure was the strongest predictor of mental health in both pregnant and postpartum women and that this relation was strongest for women with greater direct exposure [14]. Similarly, post-disaster maternal mental health was shown to predict infant social development and temperament difficulties in studies of the Quebec ice storm of 1998 and Hurricane Katrina [14]. These results are consistent with the larger body of trauma research that indicates that proximity to trauma exposure is a well-established risk factor for mental and behavioral health outcomes [15].

#### 1.1.3. Interpersonal Violence

Ahlfs-Dunn and Huth-Bocks found that infants demonstrated greater socioemotional difficulties at 12 months when mothers were exposed to interpersonal violence (IPV) during the first year following birth [16]. This association was moderated by maternal PTS symptoms, which suggests that maternal trauma severity indicators help to explain differences in infant outcomes as they pertain to maternal trauma experiences. Research also indicates that infants born to mothers who experienced IPV during pregnancy exhibit greater developmental difficulties at 10 months, including higher scores on withdrawal behaviors, greater negative affect, poorer motor coordination, less interest in play activities, higher distractibility, and more frequent crying [17].

#### 1.1.4. Posttraumatic Stress Disorder

Maternal PTSD symptoms have been shown to significantly predict emotion regulation behaviors for mothers [18]. Given that infants learn to regulate their emotions via caregiver interactions, this finding may be reflective of the poorer mother–infant interaction styles observed with trauma-exposed women. Additionally, the elevated versus non-elevated symptom grouping approach is only one way to conceptualize or quantify the severity of trauma exposure. Given that numerous studies have not confined their conceptualization of trauma solely to a PTSD diagnosis and have still demonstrated significant findings among maternal trauma experiences and infant outcomes, it may be beneficial for researchers to utilize continuous numeric variables for trauma exposure and PTS severity to better detect nuances along a continuum that may have been historically more limited by PTSD diagnostic criteria. Therefore, the present study examined trauma exposure and past-year impairment associated with traumatic event history instead of using a dichotomous variable for PTSD diagnosis.

### 1.2. Maternal Sensitivity

Maternal sensitivity is rooted in attachment theory and was initially based on Ainsworth’s study of attachment styles [19]. Specifically, Ainsworth identified three overarching behavioral patterns that emerged consistently with securely attached infants, which became known as the first operational definition of maternal sensitivity [19]. The first behavioral pattern included response to infant signals, such that signals are perceived and correctly interpreted and then promptly and appropriately responded to by the mother [19]. The second behavioral pattern included maternal tendencies to provide care that aligned with the infant’s state and mood and that were in time with the infant’s needs or desires. The third behavioral pattern involved interaction with the infant, such that the quantity of interaction was less important than the quality [19].

Maternal sensitivity has also been defined as the extent to which a mother demonstrates insightfulness about her infant’s internal experiences, responsiveness to her infant’s needs, and appropriateness in terms of maternal caregiving behaviors across contexts [20,21]. However, the pervasive inconsistencies in the ways in which maternal sensitivity is defined, assessed, and reported across studies [22,23] makes it challenging to operationalize, replicate, and extend the extant literature. Therefore, the current study utilized a well-validated standardized behavioral assessment of early mother–infant interactions to assess maternal sensitivity in a manner consistent with seminal theoretical work as well as contemporary empirical studies [5].

### 1.3. Infant Temperament

Individual differences in infant temperament have been observed from birth, and researchers have theorized that such differences are the result of both psychological and biological influences [24]. There are several models of infant temperament, but the present study will focus on infant temperament as conceptualized and defined through the psychobiological approach developed by Mary Rothbart [24,25]. Specifically, infant temperament has been previously defined from a psychobiological approach as “constitutionally [or biologically] based individual differences in reactivity and self-regulation, influenced over time by heredity, maturation, and experience” [25]. The reactivity component of the infant temperament definition involves individual differences in patterns of emotional arousal, motor activity, and attention in response to both internal and external stimuli [25]. The self-regulation component of temperament involves actions that increase or moderate such reactive tendencies [24]. Self-regulation patterns may enhance or inhibit reactivity, such as engagement in self-soothing when confronted with distressing stimuli, attentional regulation, and approach and avoidance behaviors [25]. Rothbart posited that reactivity was best captured by separating positive emotionality and negative emotionality components of temperament into two separate dimensions due to findings that individual infants could score high or low on both and that scoring high on one type did not automatically mean a low score would be obtained on the other type [24]. Rothbart’s construct of reactivity comprised two factors, surgency and negative reactivity, and self-regulation was captured with a third orienting/regulation factor [24].

### 1.4. Associations among Maternal Trauma History, Sensitivity, and Infant Temperament 

Biopsychosocial development occurs rapidly during infancy, and the bidirectional associations between maternal (e.g., trauma exposure) and infant experiences (e.g., maternal sensitivity to infants), combined with relatively stable temperamental tendencies, have important implications for long-term outcomes. In addition to empirically supported associations between maternal trauma history and maternal postnatal sensitivity across studies [1,26], researchers have supported univariate associations between each of these variables and infant temperament outcomes. Recent theoretical models also link maternal mental health, mother–infant reciprocity, and infant development in a way that suggests that maternal sensitivity to infants may mediate relations between prior maternal trauma history and infant outcomes, including important facets of temperament like reactivity and regulation [27], although no known empirical study examines this mediation hypothesis from the prenatal to infancy periods. Namely, there is a paucity of research that includes all these variables within a single study or theoretical model. Thus, our understanding of associations between early infant temperament development and individual and combined effects of these maternal risk factors is limited. Furthermore, many studies focus on the effects of one type of trauma exposure at specific timepoints, and while invaluable to the extant literature, these studies do not capture comprehensive maternal trauma history, which may be instrumental in identifying overarching patterns and mechanisms of associated infant outcomes. Additionally, studies have not disentangled the type of maternal trauma variable (e.g., exposure severity and PTS severity) that may be related most robustly to mother–infant interaction quality and infant temperament outcomes [16,28].

### 1.5. Proposed Model

Therefore, the current study fills gaps in the extant literature by examining different types of maternal trauma variables (i.e., exposure and impairment over the past year across trauma types) that stand to elucidate more robust maternal trauma predictors in a sample of women/infants followed from pregnancy through 6 months postpartum. These maternal trauma variables were investigated in relation to maternal sensitivity response patterns during behavioral observation tasks and infant temperament outcomes using a comprehensive and well-validated measure of three overarching temperament constructs (i.e., Surgency, Negative Affectivity, and Orienting/Regulation). To our knowledge, this is the first study to examine each of these constructs simultaneously via a mediation model within a sample derived from a health-provider-shortage area for mental health and primary care, wherein barriers to accessing care may confer greater risk for poor mother–infant health outcomes associated with untreated behavioral health difficulties.

### 1.6. Hypotheses

**Figure 1:**
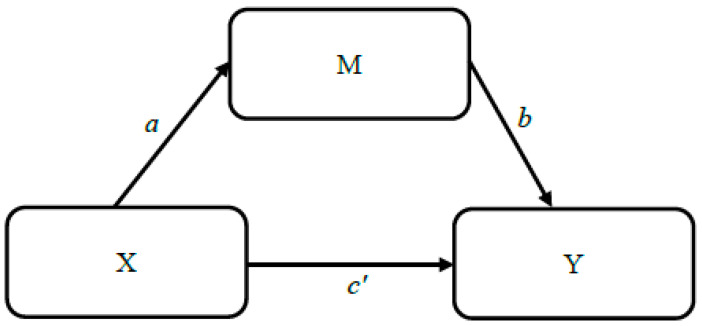
Primary analyses mediation model. Note. X = maternal trauma, M = maternal sensitivity, and Y = infant temperament; mediation model 4 adapted from “Introduction to Mediation, Moderation, and Conditional Process Analysis” [29] (p. 585).

Based on findings within the literature, we propose that maternal trauma history (as assessed by self-reported severity of trauma-associated impaired functioning over the past year and trauma event exposure) predicts maternal sensitivity (path *a*), and infant temperament (path *c′*). Additionally, maternal sensitivity is predicted to mediate relations between maternal trauma variables and infant temperament (path *ab*; see example in Figure 1 and variables included in each model/hypothesis in Table 1).

#### Hypothesis 1–3 (a–b)

Maternal sensitivity (M) mediates the relation between maternal trauma history (Hypothesis a = past-year impairment, Hypothesis b = exposure(s); X) and infant temperament (Hypothesis 1a/1b = Surgency/Reactivity, Hypothesis 2a/2b = Negative Affectivity, Hypothesis 3a/3b = Regulation/Orienting; Y), such that mothers with a greater trauma-associated impairment/exposure score will demonstrate less sensitivity toward infants, which will be related to less maternal-reported infant surgency and regulation and greater infant negative affectivity.

## 2. Materials and Methods

### 2.1. Participants 

Participants (*n* = 92) comprised mother–infant dyads (mothers between 18–35 years of age at recruitment) who participated in both the prenatal (33–37 weeks gestation) and 6-month postnatal sessions of the Infant Development and Healthy Outcomes in Mothers (IDAHO Mom) Study. While 96 of the original 125 dyads completed the postnatal session, only 92 dyads included reliable behavioral coding for the maternal sensitivity variable and therefore represented the final sample size for the present study. The majority of participants identified as White (92%), married (84%), belonging to the Church of Jesus Christ of Latter-Day Saints (64%), and having a college-level education (72%). Regarding annual household income, mothers reported income ranges of less than USD 5000 (1%), 5000–9999 (2%), 10,000–29,999 (31%), 30,000–49,999 (21%), 50,000–74,999 (28%), and 75,000–100,000 or more (13%). The sample was also largely comprised of women who reported a high level of social support satisfaction.

### 2.2. Procedure

Data were drawn from a larger study examining maternal perinatal health and infant development, entitled the Infant Development and Healthy Outcomes in Mothers (the IDAHO Mom) Study. Participants were recruited throughout southeastern Idaho (from medical centers, local businesses, schools, and libraries) via various mediums (flyers, brochures, and social media) and screened by telephone. Once eligibility was determined, mothers were scheduled for a prenatal session during the third trimester (≈33–37 weeks gestation). Mothers who reported the following conditions were deemed ineligible and excluded: gestational diabetes, toxemia, pre-eclampsia, bipolar disorder, or schizophrenia; participants younger than 18 or older than 35; mothers with multiple pregnancies; an inability to read or write in English; or exposure to a “C”, “D”, or “X” risk category medication or excessive substance use during pregnancy.

The prenatal visit consisted of consenting to participation, comprehensive interviews about health behaviors before and during pregnancy, establishment of sociodemographic characteristics, and self-report questionnaires. Participants received $30 for completion of the prenatal session. The 6-month postnatal session (±2 weeks) was scheduled 1 month after participants’ due dates, and reminder calls were scheduled to promote greater participant retention. During the 6-month session, mothers completed several Laboratory Temperament Assessment Battery sessions to assess maternal sensitivity via standardized behavioral tasks with their infants as well as clinical interviews and self-report measures. This study was approved by the Idaho State University Institutional Review Board (protocol 4191), and all participants provided written informed consent prior to engaging in research activities.

### 2.3. Measures

Maternal trauma history was assessed via self-reporting from mothers in the prenatal session, while maternal sensitivity and infant temperament were assessed at the 6-month postpartum session from coded audiovisual recordings and a self-report measure, respectively.

#### 2.3.1. Maternal Trauma History

The Trauma History Questionnaire (THQ) [30] is a 24-item Likert-type self-report measure developed to assess lifetime trauma exposure to a broad range of events [30]. The THQ contains items making up four scales that assess exposure to different types of events, including scales for crime-related events, general disaster and trauma, physical and sexual experiences, and other events not captured within the other three subscales. Each item begins with a “*yes/no*” question, indicating whether or not a specific event has been experienced, which is followed by items that quantify the number of times a specific type of event has been experienced, age at exposure, and an open-end response field to specify any other relevant details. Each item also includes two Likert-type questions (1 = *not at all* to 5 = *extremely*) to indicate how upsetting the traumatic event was at the time of exposure and how much the participant’s life had been affected by the trauma over the past year. The present study used a total exposure score across event types to best capture differences along a continuum of trauma exposure(s). Additionally, the severity of trauma-related impairment was calculated by averaging the Likert-type scale ratings across all traumatic event endorsements for the question “How much has it affected your life in the past year?”.

Test–retest reliability was measured approximately 2–3 months apart, with a sample of 25 women who reported a broad range of trauma exposure history [30]. Stability coefficients ranged from 0.51 (close person killed) to 0.91 (robbed), indicating that endorsement of specific events was fair to excellent across both THQ administrations [30]. Acceptable internal consistency reliability was found via Cronbach’s alpha calculations [31] for trauma exposure (α = 0.79) and past-year impairment across trauma domains (α = 0.85). Face validity and content validity were addressed during development of the THQ and are supported by the traumatic event dimensions agreed upon by the developers, the foundational base of previous measures, and direct relations to the DSM-IV diagnostic criterion for PTSD [30]. Furthermore, the measure has been specifically used within perinatal populations [32].

#### 2.3.2. Maternal Sensitivity

Within the current study, standardized behavioral tasks include a caregiving task, a free-play task, an orientation task, and a limitations task, to capture a wide range of mother–infant interactions. Frequency and duration values reflect whether the infant’s positive, neutral, or negative affect was met with an insensitive, moderately sensitive, or sensitive maternal response. For example, a mother who consistently and promptly engaged in effective soothing behaviors when her infant was crying would be assigned a “sensitive” classification. Conversely, a mother who consistently ignored or responded with irritation to her fussy infant would be assigned an “insensitive” classification. The coding scheme that was utilized was adapted from Leerkes and Zhou [5]. Intra-rater (across coding passes) and inter-rater coding reliability (compared to a standard created by an advanced graduate research assistant) was at least 0.80 before coding commenced [27].

**Maternal Behavior.** Maternal behavior was coded continuously to capture maternal response to infant behaviors across all standardized laboratory tasks. When mothers could not be seen in the video or were seen during times that were not meant to be coded, they were assigned a code of “0” to indicate “Uncodeable”. The mother was assigned a behavioral code of “N” for “Negative” if she demonstrated negative affect toward her infant, if she forced her own agenda on the infant, or if she laughed or smiled in response to the infant’s distress. A code of “D” was assigned for “Distracted” if the mother moved away from or abruptly ended interaction with the infant, or if the mother was uninvolved or withdrawn. A “P” code was assigned for “Persistent ineffective” if the mother persistently engaged in an ineffective response manner. An “M” code was assigned for “Monitor” if the mother was watchful of the infant but did not engage interactively with the infant. An “E” code was assigned for “Engagement” if the mother interacted with, soothed, or provided support or goal-oriented direction to the infant. An “R” code was assigned for “Routine Care” if the mother engaged in routine caregiving behavior, such as wiping the infant’s nose or straightening the infant’s clothing. If care was provided in an intrusive way, or roughly, the code was assigned an “I” for “Intrusive”.

**Infant Affect.** Infant affect was coded continuously, separately from maternal behaviors, and was based on three categories, which included *Positive* (1), *Neutral* (2), and *Negative* (3). Positive infant affect was coded when infants demonstrated positive vocalizations, smiling, wide-eyed interest, laughing, or excited body movements (i.e., clapping, moving toward stimulus). Neutral affect was coded when neither positive or negative affective behaviors were apparent. Negative affect was coded when the infant demonstrated whining, fussing, concerned facial expressions (i.e., furrowed brows, wrinkled nose), body tension, crying, screaming, or a reddened face.

Once both infant and maternal behaviors were coded, the files were merged within the INTERACT Lab Suite software (Version 2017) [33], and an automated syntax calculation was performed to create new codes based on mother–infant co-occurring behaviors [5]. These co-occurring behaviors were assigned codes based on a priori 3-point sensitivity ratings (i.e., *insensitive* = 1, *moderately sensitive* = 2, *sensitive* = 3). Reliability scores for a similar previous study were moderate and high at 6 months and 12 months, respectively (*κ* = 0.77; *κ* = 0.80) [5].

#### 2.3.3. Infant Temperament

The Infant Behavior Questionnaire–Revised Short Form (IBQ-R SF) [24,34] consists of 91 Likert-type items and was developed with the goal of capturing a broad range of nuanced infant temperament reactivity and regulation patterns. The IBQ-R SF contains three factors to measure different aspects of infant temperament, including surgency/reactivity (e.g., *“how often did your baby laugh aloud in play?”*), negative affectivity (e.g., *“how often did your baby cry or fuss before going to sleep for naps?”*), and regulation/orienting (e.g., *“when singing or talking to your baby, how often did s/he soothe”*). Developers of the IBQ-R-SF set a minimum internal consistency alpha of 0.65 for scales based on the concept that some scales were multidimensional and that a conventional cut-off value of 0.70 might unnecessarily limit the conceptual utility of findings across studies [34]. However, over 90% of Cronbach’s alpha values were greater than 0.70 for the IBQ-R-SF, which indicates generally good internal consistency. Internal consistency reliability was explored as part of the present study and was found to be acceptable [31] for each infant temperament component/domain (Surgency α = 0.87; Negative Affectivity α = 0.71; Regulation/Orienting α = 0.76).

Additionally, test–retest reliability ranged from good to excellent (0.54–0.93) across multiple time spans ranging from 2–11 months, with an average value of 0.72, which suggests strong longitudinal stability for developmental studies [34]. Convergent validity was demonstrated using the short-form IBQ–R scales in relation to the Childhood Behavior Questionnaire (CBQ), and analyses revealed that all correlations were statistically significant (*r*s = 0.17–0.34, *p* < 0.01).

#### 2.3.4. Covariates

Several covariates were investigated in reference to predictor and outcome variables, including educational attainment assessed via the Hollingshead instrument, social support (Social Support Questionnaire-6) [35], infant sex, and gestational age at birth based on mothers’ last menstrual period. 

**Educational Attainment.** Research has found positive associations between lower maternal sensitivity and lower maternal education [36,37] as well as negative associations between PTSD symptomology and educational attainment [38,39]. Additional work has shown a negative association between maternal education and infant temperament difficulties, such that mothers with lower educational attainment had infants who scored higher on activity level, duration of orienting, and fear tasks [40]. Interestingly, the direction of the association was reversed for sadness scores, such that infants of more highly educated mothers also scored higher on indicators of sadness [40]. Given associations between maternal education, predictors, and the outcome variable, educational attainment was included as a covariate in the present study. Education was assessed via the Hollingshead Four-Factor Index of Socioeconomic Status (SES), which is a widely used measure of SES that has been cited over 5000 times since development [41,42]. The four factors used to calculate SES include education, occupation, biological sex, and marital status. Limitations of the Hollingshead SES calculation methods include outdated occupational codes, shifts in education trends among women since the instrument’s development, and shifts in family roles that impact monetary resource distribution within nuclear families [43]. Despite criticisms of the Hollingshead SES, the education variable is still useful in providing a marker of socioeconomic risk. For example, education is often required for occupations that are viewed with higher prestige, and post-secondary education has historically been less accessible to low-income families [42]. Education was scored by assigning a value ranging from 1 to 7 based on educational attainment (*1* = *less than 7th grade* to *7* = *graduate professional training*).

**Social Support.** Research has shown that mothers who report higher social support are more likely to demonstrate higher maternal sensitivity [21] and that lower social support is associated with lower maternal sensitivity [37]. Additionally, low social support was significantly related to greater childhood trauma exposure and poorer mental health compared with healthy controls [44]. Additional research has shown that steeper diurnal cortisol rhythms are positively related to social support in a sample of adult men and women [45], which may be indicative of a more efficient, adaptive stress response that is dependent on social support availability. 

Social support was evaluated via the Social Support Questionnaire-6 (SSQ-6) [35]. The SSQ-6 assesses participant perceptions of social support relationships. Participants are asked to list up to nine people who can be depended upon for social support across a variety of contexts in six separate items. Participants are also instructed to specify their relation to the people listed. This includes people (1) who the participant can count on to be dependable when help is needed, (2) who can help the participant to feel relaxed when under pressure, (3) who wholly accept the participant at their worst/best points, (4) who can be counted on to care about the participant regardless of the situation, (5) who can help the participant to feel better when “down in the dumps”, and (6) who can be counted on to console the participant when upset. Participants then rate satisfaction level with social support in each context on a 6-point Likert-type scale (*1* = *very satisfied* to *6* = *very dissatisfied*). There are two common scoring methods for the SSQ-6, which include the SSQ Number Score (SSQN) and the SSQ Satisfaction Score (SSQS). The SSQN is derived by calculating the mean of the total number of people listed for all six items. The SSQS score is calculated by averaging the satisfaction scores for all six items. The internal consistency alpha coefficients for both number and satisfaction scores range from 0.90 to 0.93 [35]. 

Only the SSQS score will be utilized as a covariate within the present study, due to the inherent implications of social support quality. High satisfaction ratings of social support relationships, regardless of the number of people included in the network, are more likely to serve as a protective factor against adversity, whereas high numbers of people do not necessarily indicate a high-quality social support network. Therefore, the construct of social support satisfaction is more meaningful within the present study and will be the sole covariate indicator of social support.

**Infant Sex.** Findings from an empirical review suggest that male fetuses may be more sensitive to maternal prenatal cortisol exposure (which is associated with maternal trauma) than female fetuses [46,47], which is due to hormonal differences that emerge during sex differentiation. Additional research indicates that while females may adapt to maternal prenatal cortisol exposure more efficiently than males in early development, females may experience more adverse long-term impacts in terms of anxious behaviors and greater negative affectivity [48,49]; more research is needed to confirm when temporal differences manifest across development. These findings outline a need to examine infant sex as a potential covariate in the current study. Infant sex was determined from maternal self-reporting during the 6-month postnatal visit via a single item on the IBQ-R-SF, *“What is your baby’s sex?”* [34]. Female infants were assigned a code of “*1*”, and male infants were assigned a code of “*0*”. 

**Gestational Age at Birth.** Research has shown infant gestational age at birth to significantly predict maternal sensitivity [21]. Specifically, mothers who delivered at 37 weeks gestation or less were more likely to be more sensitive toward their infants [21]. Given that mothers were recruited between 33–37 weeks gestation in the present study, gestational age at birth will be included as a covariate. Mothers’ last menstrual period (LMP) was deemed to be the best method for use in the present study to assess gestational age at birth [50,51]. Following data collection, gestational age calculations were quality-checked by one undergraduate research assistant and one graduate research assistant by cross-referencing participant delivery dates in data tracking files and by replicating calculations to ensure accuracy.

### 2.4. Quantitative Analyses

A G*Power *a priori* power analysis was conducted to determine the sample size needed to achieve a power of 0.80 in this study. A least-squares linear multiple regression with three predictors, one outcome variable, and up to three covariates was performed and was based on a medium effect size (*f*^2^ = 0.15) [52] (p. 412) and a two-tailed *p*-value of 0.05. Results indicated that a sample size of 77 was needed. Previous research [53] indicates that a sample size of 71 is necessary to attain 0.80 power with bias-corrected bootstrapping assessment methods in mediation models with medium effect sizes (*d* = 0.39). Therefore, the present study sample size (*n* = 92) was deemed to yield sufficient power to proceed with the data analysis plan. Analyses were conducted utilizing IBM SPSS Statistics (Version 27) and Hayes PROCESS Macro via mediation modeling [29] (p. 585).

## 3. Results

### 3.1. Descriptive Statistics and Covariates

Means and standard deviations were calculated for each primary variable and the covariates. Mothers endorsed exposure to an average of approximately three types of traumatic events (*M* = 2.7, *SD* = 0.3), which ranged from 0 to 16 event types endorsed across the sample. Mothers’ scores on impairment across trauma domains over the past year averaged between “1 = *not at all*” and “2 (no qualitative descriptor)” across trauma domains (*M* = 1.5, *SD* = 0.2) and with sample responses ranging across the entire Likert-type scale from “1 = *not at all*” to “5 = *extremely*”. Maternal sensitivity coding resulted in an approximate average of 94.4 instances of sensitive responding across approximately 20 total minutes of behavioral tasks (*M* = 94.4 s, *SD* = 2.8 s). Regarding infant temperament, the Surgency (*M* = 5.0, *SD* = 0.1) and Regulation/Orienting (*M* = 5.2, *SD* = 0.05) factors resulted in average values indicative of associated behaviors occurring approximately “*more than half the time*”. The Negative Affectivity (*M* = 3.1, *SD* = 0.7) factor resulted in an average value indicative of associated behaviors occurring “*less than half the time*”.

Regarding covariate descriptive statistics, infant sex frequencies revealed that of the 96 infants who completed the 6-month postnatal session, 49% were female (*n* = 47) and 51% were male (*n* = 49). Infant gestational age at birth averaged approximately 39 weeks (*M* = 39.4 weeks, *SD* = 0.1 weeks). Mothers’ educational attainment scale scores indicated an average education rating commensurate with a standard college degree (*M* = 5.3, *SD* = 0.1). Social support quality, as measured with the SSQS, revealed an average “*very satisfied*” rating (*M* = 5.4, *SD* = 0.1).

Pearson product-moment correlation coefficients were calculated to determine if statistically significant relationships existed amongst covariates and predictor and outcome variables. Infant sex assigned at birth and Negative Affectivity were negatively associated (*r* = −0.21, *p* = 0.039). A follow-up independent sample *t*-test revealed a statistically significant difference (*t* (94) = −2.1, *d* = −0.43, *p* = 0.04, 95% CI [−0.52, −0.01]) between boys and girls, such that girls (*M* = 3.22, *SD* = 0.58) scored higher on Negative Affectivity than boys (*M* = 2.95, *SD* = 0.66). Therefore, infant sex assigned at birth was included as a covariate in the primary mediation models containing the outcome variable of Negative Affectivity. No other covariate relations were statistically significant; thus, social support quality, educational attainment, and gestational age at birth were not included as covariates in the primary analyses.

### 3.2. Mediation Models

#### 3.2.1. Model 1 (Hypothesis 1a)

The overall mediation model predicting the Surgency factor of infant temperament via past-year trauma-related impairment and maternal sensitivity was not statistically significant (*F*[1, 90] = 1.28, *R*^2^ = 0.01, *p* = 0.26). The *a* (*b* = 8.87, *t*[92] = 0.89, *SE* = 10.01, *p* = 0.38), *b* (*b* = 0.0001, *t*[92] = 0.04, *SE* = 0.002, *p* = 0.97), and *c′* (*b* = −0.23, *t*[92] = −1.13, *SE* = 0.20, *p* = 0.26) paths were not statistically significant. The indirect effect of past-year trauma-related impairment on Surgency via maternal sensitivity was also not statistically significant (*b* = 0.0008, *SE* = 0.03, 95% CI [−0.04, 0.07]).

#### 3.2.2. Model 2 (Hypothesis 1b)

The overall mediation model predicting the Surgency factor of infant temperament via trauma exposure and maternal sensitivity was not statistically significant (*F*[1, 90] = 0.85, *R*^2^ = 0.009, *p* = 0.36). The *a* (*b* = −3.11, *t*[92] = −0.35, *SE* = 8.95, *p* = 0.73), *b* (*b* = −0.0001, *t*[92] = −0.028, *SE* = 0.002, *p* = 0.98), and *c′* (*b* = 0.16, *t*[92] = 0.91, *SE* = 0.18, *p* = 0.36) paths were not statistically significant. Likewise, the indirect effect of trauma exposure on Surgency via maternal sensitivity was also not statistically significant (*b* = 0.0002, *SE* = 0.02, 95% CI [−0.04, 0.03]).

#### 3.2.3. Model 3 (Hypothesis 2a)

The overall mediation model predicting the Negative Affectivity factor of infant temperament via past-year trauma-related impairment and maternal sensitivity while controlling for infant sex was not statistically significant (*F*[2, 89] = 1.95, *R*^2^ = 0.042, *p* = 0.15). The *a* (*b* = −1.01, *t*[92] = −0.018, *SE* = 5.72, *p* = 0.86)*, b* (*b* = −0.26, *t*[92] = −1.94, *SE* = 0.13, *p* = 0.6), and *c′* (*b* = 0.017, *t*[92] = 0.07, *SE* = 0.24, *p* = 0.94) paths were not statistically significant when controlling for infant sex. The indirect effect of past-year trauma-related impairment on Negative Affectivity via maternal sensitivity was also not statistically significant (*b* = 0.012, *SE* = 0.03, 95% CI [−0.07, 0.07]).

#### 3.2.4. Model 4 (Hypothesis 2b)

The overall mediation model predicting the Negative Affectivity factor of infant temperament via trauma exposure and maternal sensitivity (while controlling for infant sex) was not statistically significant (*F*[2, 89] = 1.97, *R*^2^ = 0.04, *p* = 0.15). The *a* (*b* = −1.21, *t*[92] = −0.21, *SE* = 5.74, *p* = 0.83), *b* (*b* = −0.26, *t*[92] = −1.96, *SE* = 0.13, *p* = 0.05), and *c′* (*b* = −0.56, *t*[92] = 0.27, *SE* = 0.21, *p* = 0.79) paths were not statistically significant. The indirect effect of trauma exposure on Negative Affectivity via maternal sensitivity was also not statistically significant (*b* = −0.004, *SE* = 0.02, 95% CI [−0.04, 0.06]).

#### 3.2.5. Model 5 (Hypothesis 3a)

The overall mediation model predicting the Regulation/Orienting factor of infant temperament via past-year trauma-related impairment and maternal sensitivity was not statistically significant (*F*[1, 90] = 2.35, *R*^2^ = 0.025, *p* = 0.13). The *a* (*b* = 8.87, *t*[92] = 0.89, *SE* = 10.01, *p* = 0.38), *b* (*b* = −0.001, *t*[92] = −0.51, *SE* = 0.002, *p* = 0.61), and *c′* (*b* = −0.26, *t*[92] = −1.47, *SE* = 0.17, *p* = 0.15) paths were not statistically significant. The indirect effect of past-year trauma-related impairment on Regulation/Orienting via maternal sensitivity was also not statistically significant (*b* = −0.008, *SE* = 0.02, 95% CI [−0.06, 0.04]).

#### 3.2.6. Model 6 (Hypothesis 3b)

**Figure 2:**
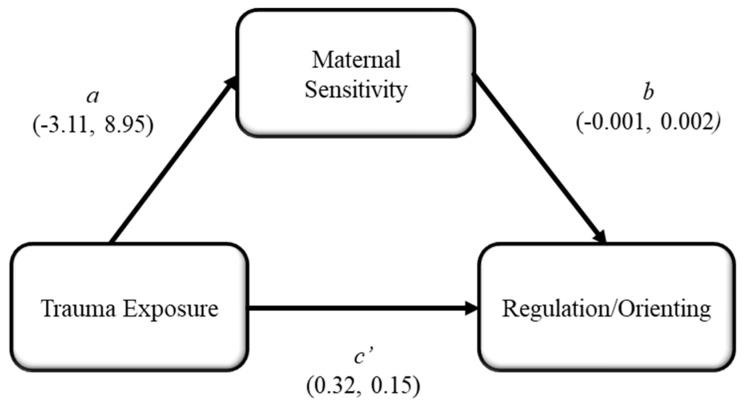
Model 6 Hypothesis 3b. Note: Unstandardized beta coefficient and standard deviation results for each path are (*b*, *SD*).

The overall mediation model predicting the Regulation/Orienting factor of infant temperament via trauma exposure and maternal sensitivity was statistically significant (*F*[1, 90] = 4.46, *R*^2 =^ 0.05, *p* = 0.04; see Figure 2). Both the *a* (*b* = −3.11, *SE* = 8.95, *t*[92] = −0.35, *p* = 0.73) and *b* paths were not statistically significant (*b* = −0.001, *SE* = 0.002, *t*[92] = −0.58, *p* = 0.56). However, there was also a statistically significant direct effect of trauma exposure on Regulation/Orienting while considering maternal sensitivity in the model (*b* = 0.32, *SE* = 0.15, *t*[92] = 2.08, *p* = 0.04). The indirect effect of trauma exposure on Regulation/Orienting via maternal sensitivity was not statistically significant (*b* = 0.003, *SE* = 0.017, 95% CI [−0.03, 0.04]).

## 4. Discussion

### 4.1. Mediation Models

Results from the present study did not support mediation through maternal sensitivity for any of the primary analysis models. There may be a few contributing factors to this finding, which include potential range restriction and ceiling effects within the present sample. Notably, the sample majority indicated approximately no impairment associated with past trauma, which suggests that there may not have been enough variance in trauma impairment scores to detect potential effects that may be present in mothers with varying levels of posttraumatic impairment. The sample was also largely comprised of well-educated, married women with a high level of social support satisfaction.

### 4.2. Infant Covariates

#### 4.2.1. Infant Gender Differences

There were no statistically significant correlations among covariates and primary study variables, except for the positive association found between infant sex assigned at birth and negative affectivity [46,47]. Analyses revealed that infants assigned female at birth scored higher in negative affect than infants assigned male at birth. This replicates existing research literature [48] and may be due to differences in maternal perceptions based on infant sex assigned at birth or the sex-specific effects of other prenatal factors (e.g., exposure to in utero cortisol). Despite perceived differences in negative affect based on sex assigned at birth, there were no statistically significant findings in direct or indirect pathways in the models that examined negative affectivity when infant sex assigned at birth was included as a covariate.

#### 4.2.2. Maternal Trauma

Additionally, despite the wide range of trauma exposure endorsements across trauma event types, average impairment scores were only just above “*not at all*”, which suggests that overall impairment was not elevated to the degree that mothers were significantly affected by their overall past trauma experiences. This finding is consistent with research that has shown that most trauma victims do not develop clinically elevated PTS [54].

#### 4.2.3. Maternal Trauma and Infant Temperament

A direct effect and a positive association were found between maternal trauma exposure and the infant temperament Regulation/Orienting factor. No other direct or indirect effects were observed. It is important to consider the direction of the relation between maternal trauma exposure and infant regulation, which was in opposition to the anticipated direction of effect, where greater trauma exposure would predict decreased infant regulation behaviors. Sample characteristics may help to explain these results. Specifically, prior research has demonstrated positive associations between maternal sensitivity and social support [21,37] and negative associations between maternal trauma and social support [44], and the present sample largely indicated a “*very high*” satisfaction rating for social support quality. Research has also found positive associations between maternal sensitivity and maternal education [36,37] and negative associations between PTSD symptomology and education [38,39], and the majority of the present study sample reported having at least a college education. Taken together, it may be that mothers with the protective factors present within this sample (e.g., “*very high*” social support satisfaction, endorsed religious affiliation, and college education) were better enabled to adjust in an adaptive manner following traumatic experiences [38,39] and were therefore better enabled to develop sensitive maternal behaviors with their infants [21,36,37] and protectively buffered against the effects and/or development of clinically elevated PTS. This aligns with contemporary research supporting constructs like posttraumatic growth and protective and compensatory experiences that build resilience [55]. Collectively, these patterns may have supported development of greater infant regulation ability [3,56]. These are prospective hypotheses, and further research should address these potential relations to broaden our understanding of the potential effects and relationship directions between maternal trauma variables and infant temperament outcomes.

### 4.3. Study Limitations and Future Directions

It is important to consider that the IDAHO Mom Study was not primarily designed for trauma research, and it is possible that participants who may have had more diverse trauma-related experiences were excluded from the study upon eligibility screening due to endorsement of associated risk factors (e.g., serious mental health concerns). Moreover, while the continuous trauma variables provided an estimate of overall trauma exposure and post-trauma impairment, revictimization and polyvictimization were not distinguished from one another and could have elucidated outcome differences if assessed separately [57,58]. Additionally, given that maternal trauma history was assessed in the prenatal period and maternal sensitivity and infant temperament were both assessed in the 6-month postpartum session, it is possible that trauma exposure and impairment across trauma domains could have changed between sessions and therefore may not have comprehensively captured variable relationships. In addition, the present study hypotheses predicted linear relationships among primary variables; however, evidence suggests that such relations may not be linear (e.g., cumulative risk modeling in trauma) [59]. It may be that linear modeling of maternal trauma, maternal sensitivity, and infant temperament variables did not capture a full range of potential effects among variables and does not account for the complexity of individual differences in cumulative risk associated with maternal trauma exposure.

While the aim of the present study was to analyze mothers’ lifetime trauma exposure and impairment scores across trauma types via continuous variables, effects may not have been detected due to not controlling for the specific types of trauma (e.g., IPV, CT, disaster), the recency of traumatic events, and proximity (e.g., witnessing an event versus hearing about an event), which have demonstrated statistically significant results among primary variables in previous work [11,12,16,17,28,60]. Notably, these variables are also associated with biological stress responses (e.g., hypothalamic–pituitary–adrenal (HPA) axis), which has important implications for mother–infant stress physiology during the gestational period [8,46,61]. Maternal cortisol is also related to maternal sensitivity and infant temperament outcomes [46,62]. HPA axis functioning was not considered within the scope of the present study and may provide greater insight into primary variable relationships in future work.

Given that the present sample largely demonstrated characteristics that are associated with greater maternal sensitivity (e.g., high social support satisfaction and college education), our ability to detect significant findings may have been limited. It may also be that mothers were responding more sensitively than usual during the one-time live observation method due to knowing that they were being observed by the research assistants and awareness that they were being recorded.

Future studies should consider examining additional trauma variables (e.g., event type, timing, recency) and including sensitive, insensitive, and ambiguous maternal responding within the maternal sensitivity construct. Additionally, previous research has indicated that specific subscales (e.g., falling reactivity, activity, cuddliness) within the infant temperament factors (e.g., Surgency, Negative Affectivity, and Surgency) of the IBQ-R-SF are significantly related to both maternal trauma and maternal sensitivity [3,11,63]. It may be that further analysis of the more specific infant temperament behaviors defined by these subscales would yield greater insight into potential differences among primary variable relations within the IDAHO Mom Study sample.

Further, HPA axis alterations due to trauma exposure often persist long after a traumatic event has transpired, particularly in women [1], and are often unresolved prior to pregnancy [6]. Therefore, inclusion of maternal prenatal cortisol release as a predictor in future research may provide a more comprehensive model of psychobiological infant temperament risk in relation to maternal trauma history and maternal sensitivity behaviors.

Finally, results from this study highlight the need for future research to explore disparities among mother–infant dyads with a more diverse range of social support quality, education, marital status, religious affiliation, and offspring gestational age at birth. A large portion of respondents to the study recruitment advertisements declined to participate due to commute and time commitment concerns. A more diverse sample, inclusive of single mothers with less education and lower social support quality, who reside in rural versus urban areas and have more limited income resources, may be best recruited in future research by conducting study sessions within the subjects’ homes to ease the burden of transportation and time concerns.

## 5. Conclusions

The present study addressed gaps in the literature by examining the unique and combined associations of maternal trauma and sensitivity in relation to infant temperament reactivity and regulation outcomes. Additionally, the present study utilized well-validated and reliable measures of primary variables with subscales and factors that demonstrated acceptable internal consistency with the present sample. While the mediation hypotheses were not statistically significant, the results add to the extant literature by providing insight into maternal trauma, maternal sensitivity, and infant temperament outcomes within a sample of prenatal women and 6-month-old infants who have access to greater social support quality and education in a federally designated underserved primary care and mental healthcare provider shortage area.

## Figures and Tables

**Table 1:** Summary of mediation model hypothesis 1–3 (a–b).

Model	Maternal Sensitivity (M)	Maternal Trauma (X)	Infant Temperament (Y)
Hypothesis 1a	Total Frequency	Past Year Impairment	Surgency/Reactivity
Hypothesis 1b	Total Frequency	Exposure	Surgency/Reactivity
Hypothesis 2a	Total Frequency	Past Year Impairment	Negative Affectivity
Hypothesis 2b	Total Frequency	Exposure	Negative Affectivity
Hypothesis 3a	Total Frequency	Past Year Impairment	Regulation/Orienting
Hypothesis 3b	Total Frequency	Exposure	Regulation/Orienting

Note. M = mediation variable, X = predictor variable, Y = outcome variable. M was proposed to mediate the relationship between X and Y.

## Data Availability

The data presented in this study may be available on request from the corresponding author. The data are not publicly available due to specific ethical and privacy considerations.
